# Requirement of preoperative blood typing for cholecystectomy and appendectomy: a systematic review

**DOI:** 10.1007/s00423-022-02600-x

**Published:** 2022-07-02

**Authors:** Michael G Fadel, Ishaan Patel, Lawrence O’Leary, Nebil Behar, James Brewer

**Affiliations:** Department of General Surgery, Chelsea and Westminster Hospital, 369 Fulham Road, London, SW10 9NH UK

**Keywords:** Blood typing, Group and save testing, Type and screen, Cholecystectomy, Appendectomy, Blood transfusion

## Abstract

**Purpose:**

Blood typing, or group and save (G&S) testing, is commonly performed prior to cholecystectomy and appendectomy in many hospitals. In order to determine whether G&S testing is required prior to these procedures, we set out to evaluate the relevant literature and associated rates of perioperative blood transfusion.

**Methods:**

Studies from January 1990 to June 2021 assessing the requirement of preoperative G&S testing for elective or emergency cholecystectomy and appendectomy were retrieved from MEDLINE, EMBASE and CINAHL databases. The search was performed on 6th July 2021 (PROSPERO registration number CRD42021267967). Number of patients, co-morbidities, operation performed, number of patients that underwent preoperative G&S testing, perioperative transfusion rates and financial costs were extracted.

**Results:**

We initially screened 194 studies of which 15 retrospective studies, a total of 477,437 patients, specifically met the inclusion criteria. Ten studies reported on cholecystectomy, two studies on appendectomy and three studies included both procedures. Where reported, a total of 177,539/469,342 (37.8%) patients underwent preoperative G&S testing with a perioperative transfusion rate of 2.1% (range 0.0 to 2.1%). The main preoperative risk factors associated with perioperative blood transfusion identified include cardiovascular co-morbidity, coagulopathy, anaemia and haematological malignancy. All 15 studies concluded that routine G&S is not warranted.

**Conclusion:**

The current evidence suggests that G&S is not necessarily required for all patients undergoing cholecystectomy or appendectomy. Having a targeted G&S approach would reduce delays in elective and emergency lists, reduce the burden on the blood transfusion service and have financial implications.

**Supplementary Information:**

The online version contains supplementary material available at 10.1007/s00423-022-02600-x.

## Introduction

Group and save (G&S) testing is frequently performed prior to surgery to check blood type and screen for irregular antibodies. Currently, there are no universally accepted national or international guidelines that recommend which patients or procedural factors warrant routine preoperative G&S screening for emergency or elective laparoscopy.

The National Institute for Health and Care Excellence (NICE), UK, produced guidelines in 2016 to standardise and reduce unnecessary testing prior to elective surgery. Recommended tests were stratified by the complexity of surgery and patient co-morbidities; however, advice on G&S testing was not included [1]. The decision to perform G&S testing usually relies on the clinical judgement of surgical and anaesthetic teams—or commonly in elective surgery, on nursing staff running preoperative clinics.

The French Society of Anaesthesiology and Intensive Care (SFAR) published guidelines in 2012 advising against the routine use of G&S testing if the risk of bleeding is deemed low [2]. In these guidelines, the authors do not further define this; however, a subsequent study by the same authors specified laparoscopic cholecystectomy as an example of a surgical procedure that does not require routine preoperative G&S testing [3]. Possibly owing to the lack of clarity in the original guidelines or due to the fact that they were never published in English, these recommendations have not become universal as of yet.

Major vascular injury during laparoscopic surgery that would necessitate immediate blood transfusion includes damage to the aorta and direct branches, the vena cava and its major tributaries, as well as the portal vein. Laparoscopic entry is associated with a low incidence of major vascular injury [4–6]. Molloy et al. [7] performed a meta-analysis which demonstrated that vascular injury rates with the Veress needle and open technique are 0.004% and 0.001% respectively. Intraoperative bleeding is relatively rare with laparoscopic surgery. For example, in a study by Z’graggen et al. [8] of 10,174 laparoscopic cholecystectomies, the intraoperative bleeding rate was 1.97%. In a Finnish series of 1581 laparoscopic cholecystectomies [9, 10], the reported incidence of bleeding complications was 1.1% with a reoperation rate of 0.5% in these cases.

In the rare case of major vascular injury during laparoscopic surgery, urgency of blood products means there is potentially little added value in G&S over the use of O negative blood. The time taken to procure cross-matched blood following G&S (or to even receive group-specific blood) would often be detrimental in such cases [11]. A haemorrhage protocol should be initiated resulting in blood products such as O negative blood, fresh frozen plasma, platelets, cryoprecipitate and tranexamic acid being immediately available.

G&S screening is still being performed prior to cholecystectomy and appendectomy operations in many hospitals. There is a need for clear recommendations regarding the necessity and selectivity of G&S testing in common emergency and elective laparoscopic surgery. To help achieve this, we sought to perform a systematic review to evaluate G&S testing prior to cholecystectomy and appendectomy, and perioperative blood transfusion rates. This in turn would allow us to assess the need for G&S testing and perioperative risk factors for blood transfusion in order to improve patient outcomes, hospital resources and efficiency.

## Methods

### Search strategy

A systematic review of the literature concerning G&S testing prior to cholecystectomy and appendectomy was conducted according to the protocol recommended by the Cochrane collaboration [12]. MEDLINE, EMBASE and CINAHL databases were searched for studies published between January 1990 and June 2021 in the English language. The search was performed on 6^th^ July 2021. The following medical subject headings (MeSH) and keywords were used: ‘blood type’, ‘group and save’, ‘group and screen’, ‘group and antibody’, ‘type and screen’, ‘cholecystectomy’, ‘appendectomy’, ‘appendicectomy’, ‘laparoscopy’, ‘elective procedure’, ‘emergency procedure’ and 'blood transfusion'. We also performed a manual search of the references from selected articles which related to our research to identify additional relevant studies. The work was registered in the PROSPERO database for systematic reviews in August 2021 (CRD42021267967). The study was reported in line with PRISMA (Preferred Reporting Items for Systematic Reviews and Meta-Analyses) and AMSTAR-2 (Assessing the methodological quality of systematic reviews) guidelines [13].

### Study selection and inclusion criteria

Studies were selected if they met the following criteria: retrospective or prospective cohort studies, case–control studies or cross-sectional studies. The studies chosen had to be specifically related to G&S testing in cholecystectomy or appendectomy. Studies reporting these procedures as either elective or emergency in adults and paediatric patients were included. Studies that reported on the perioperative blood transfusion rate but did not specifically comment on the number of patients that underwent G&S testing for either cholecystectomy or appendectomy procedures were also included if they reached a conclusion regarding the necessity of G&S testing. Conference abstracts, case series and studies lacking relevant outcomes were excluded from the systematic review.

### Outcomes of interest and endpoints

Studies reporting the requirement of preoperative G&S testing in elective or emergency cholecystectomy and appendectomy were selected. Number of patients, patient demographics/co-morbidities, type of operation performed, number of patients that underwent preoperative G&S testing, complications, definition of perioperative blood transfusion, perioperative blood transfusion rate and financial costs were extracted where reported.

Perioperative blood transfusion was defined as per the included studies and the definition for each individual study was recorded in summary tables. For cholecystectomy procedures, perioperative blood transfusion was defined as either given during the admission, intraoperatively or within 48 h of the procedure. For appendectomy procedures, the definition of perioperative blood transfusion differed across the studies: on the day of or after the procedure, during the index admission or within 30 days of the index admission.

### Data extraction and quality assessment

The titles and abstracts were assessed, by one of the authors (MGF), against the inclusion and exclusion criteria, arriving at a final list of articles. Each included manuscript was read to determine ultimate inclusion in the final analysis. A second reviewer (IP) confirmed that the final selected manuscripts met the inclusion criteria. From the manuscripts, the following information was extracted: author names, year of publication, title, country of origin, study design, patient selection criteria (e.g. age), analysis method, outcome measures, results and follow-up.

The quality of the included studies was assessed by one author (LO’L) using the Joanna Briggs Institute Critical Appraisal Checklist for Studies Reporting Prevalence Data [14], a well-established and validated system for appraising observational studies reporting prevalence data. This nine-point checklist allows for an objective measure of risk of bias of reported prevalence data. Each criterion has a binary score: ‘Yes’ if it is met; otherwise ‘No’. Appointed scores were checked by the other authors (MGF and IP) and any disagreements resolved through discussion. The quality of a study was deemed ‘acceptable’ if at least seven of the criteria were met, a cut-off that is widely accepted [15–17].

Question 3 of the checklist, which relates to whether the sample size was adequate, was deemed to have been met if the number of participants in the study exceeded 380 participants. This was derived from the power calculation described by Naing and colleagues [18]:

$$n=\frac{{Z}^{2}P\left(1-P\right)}{{d}^{2}}$$

where.

*n*: sample size,
*Z*: *Z* statistic for a level of confidence (set at 1.96 for this review),
*P*: expected prevalence or proportion (set at 0.01 for this review), and
*d*: precision (set at 0.01 for this review).

### Statistical analysis

The studies were assessed for information regarding the number and percentage of patients that underwent preoperative G&S testing, blood transfusion rates, patient and operative factors for those that received a blood transfusion. Financial costs were also calculated for both the study cohort and per annum (£). The mean, median, range and standard deviation were calculated where applicable. The data was summarised in tables, also highlighting any missing data for the individual study. Information on clinical practice along with established guidelines on the use of G&S preoperatively prior to laparoscopic surgery was also reviewed.

## Results

**Fig. 1:**
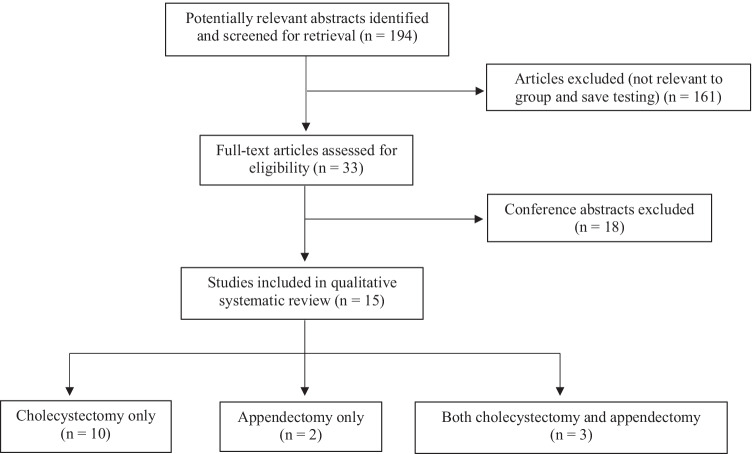
PRISMA flow diagram of studies in this systematic review

The literature search identified 194 studies. All the abstracts were screened and 15 full-text articles [11, 19–32] strictly met the study inclusion criteria—a total of 477,437 patients. All were retrospective studies: 10 studies [11, 19, 21, 23, 25–30] reviewed the necessity of G&S for cholecystectomy procedures only, two studies [31, 32] on appendectomy procedures only and three studies [20, 22, 24] evaluated both procedures. A PRISMA [33] flowchart of the section process for this study is presented in Fig. 1. Only six [19, 20, 24, 26, 31] out of the 15 studies had complete data on age and gender, and therefore, this was not included in the summary analysis.

### Cholecystectomy procedures

The studies reporting on outcomes of patients who underwent cholecystectomy, proportion of patients that underwent G&S testing and received perioperative blood transfusion and the quality of the study are summarised in Table 1. All studies were deemed acceptable according to the Joanna Briggs Institute Critical Appraisal Checklist for Studies Reporting Prevalence Data. The scoring of each study is shown in supplementary material Table S1.

**Table 1:** Summary of studies assessing number of patients who underwent group and save testing and perioperative blood transfusion rates in cholecystectomy procedures

Authors, year	Study type, institution and location	Type of cholecystectomy procedure performed, *n*	Total number of patients who underwent cholecystectomy, *n*	Preoperative G&S testing, *n* (%)	Definition of perioperative blood transfusion	Patients who received perioperative blood transfusion stratified by type of procedure, *n* (%)	Quality of study*
Barrett-Lee et al. [20], 2018	Retrospective study, single hospital trust in the UK	Emergency laparoscopic: 23 Emergency converted-to-open: 2	25	514 out of 562 (91.5)†	Not defined	Total: 0 (0.0)	Acceptable
Beloeil et al. [21], 2017	Retrospective study, hospitals nationwide in France	Emergency and elective laparoscopic‡	459,615	170,749 (37.2)	Within 48 h of procedure	Total: 9652 (2.1)	Acceptable
Blank et al. [22], 2018	Retrospective study, single hospital in Australia	Emergency and elective laparoscopic: 1110 Emergency and elective converted-to-open: 10	1120	–	During index admission	Total: 18 (1.6) Emergency and elective laparoscopic: 16 (1.4) Emergency and elective converted-to-open: 2 (20)	Acceptable
Fong et al. [23], 2021	Retrospective study, single hospital trust in the UK	Emergency and elective laparoscopic: 962 Emergency and elective converted-to-open: 40	1002	896 (89.4)	Not defined	Total: 12 (1.2) Emergency and elective laparoscopic: 6 (0.6) Emergency and elective converted-to-open: 6 (15)	Acceptable
Ghirardo et al. [24], 2010	Retrospective study, single hospital in the USA	Emergency laparoscopic: 683 Emergency open: 28 Elective laparoscopic: 454 Elective open: 2	1167	–	On day of or day after procedure	Total: 5 (0.4) Emergency laparoscopic: 4 (0.6) Emergency open: 1 (3.6) Elective laparoscopic: 0 (0.0) Elective open: 0 (0.0)	Acceptable
Hack-Adams et al. [25], 2015	Retrospective study, single hospital trust in the UK	Elective laparoscopic	53	17 (32.0)	Intraoperative	Total: 0 (0.0)	Acceptable
Hamza et al. [11], 2015	Retrospective study, three hospitals in the UK	Elective laparoscopic	913	913 (100)	Not defined	Total: 8 (0.9)	Acceptable
Li and Low [26], 2020	Retrospective study, single hospital trust in the UK	Emergency laparoscopic: 11 Elective laparoscopic: 482	493	483 (98.0)	Not defined	Total: 2 (0.40) Emergency laparoscopic: 0 (0.0) Elective laparoscopic: 2 (0.4)	Acceptable
Lin et al. [27], 2006	Retrospective study, single hospital in Taiwan	Elective laparoscopic	71	6 (8.5)	On day of procedure	Total: 1 (1.4)	Acceptable
Quinn et al. [28], 2011	Retrospective study, hospitals across a Scottish region	Emergency and elective, laparoscopic and open‡	4462	2916 (65.4)	Not defined	Total: 48 (1.1)	Acceptable
Tandon et al. [29], 2017	Retrospective study, single hospital trust in the UK	Elective laparoscopic	2079	934 (44.9)	Not defined	Total: 12 (0.6)	Acceptable
Thomson et al. [30], 2016	Retrospective study, single hospital trust in the UK	Emergency and elective laparoscopic‡	293	264 (90.1)	Not defined	Total: 0 (0.0)	Acceptable
Usal et al. [19], 1999	Retrospective study, single hospital in the USA	Emergency and elective laparoscopic: 2589 Emergency and elective open: 603	3192	–	Not defined	Total: 45 (1.4) Emergency and elective laparoscopic: 12 (0.5) Emergency and elective open: 33 (5.5)	Acceptable

^*^Quality assessed using the Joanna Briggs Institute Critical Appraisal Checklist for Studies Reporting Prevalence Data [14]: see supplementary material Table S1 for details of these assessments. ‡Number of each type of procedure not specified; †data not subdivided between cholecystectomy and appendectomy therefore these figures include all procedures in the study (*n* = 562); – exact number unknown. *G&S*, group and save; *UK*, United Kingdom; *USA*, United States of America

A total of 474,485 patients underwent cholecystectomy. Laparoscopic cholecystectomy was completed in at least 469,338 (98.9%) patients. Ten [11, 20, 21, 23, 25–31] out of 13 studies recorded the number of patients that underwent preoperative G&S testing. Data was extractable in nine of these studies: a total of 177,178/468,981 (37.8%, range 8.5–100%) patients. Only 25 patients had a cholecystectomy in the Barrett-Lee et al. [20] study and the number of patients that had a G&S test prior to this specific procedure was not reported. Beloeil et al. [21] had the largest cohort of patients that underwent laparoscopic cholecystectomy of 459,615 patients, with 170,749 (37.2%) patients completing preoperative G&S testing. The other eight studies that reported G&S testing had 6429/9366 (68.6%) patients with G&S screening. A total of 9803 (2.1%, range 0.0–2.1%) patients received a perioperative blood transfusion for cholecystectomy.

### Appendectomy procedures

Summaries of the data reported on appendectomies are presented in Table 2. A total of 2952 patients underwent this procedure. Two [19, 32] of the five studies reported preoperative G&S testing rates. Three hundred and sixty-one (100%) patients had a valid G&S test prior to appendectomy in the Magowan et al. [32] study. In the Barrett-Lee et al. [20] study, a total of 514 (91.5%) out of 562 patients that underwent a general surgical procedure had prior G&S testing, of which 494 patients had an appendectomy.

**Table 2:** Summary of studies assessing number of patients who underwent group and save testing and perioperative blood transfusion rates in appendectomy procedures

Authors, year	Study type, institution and location	Type of appendectomy procedure performed, n	Total number of patients who underwent appendectomy, *n*	Preoperative G&S testing, *n* (%)	Definition of perioperative blood transfusion	Patients who received perioperative blood transfusion stratified by type of procedure, *n* (%)	Quality of study*
Barrett-Lee et al. [20], 2018	Retrospective study, single hospital trust in the UK	Emergency laparoscopic: 446 Emergency converted-to-open: 23	469	514 out of 562 (91.5)†	Not defined	Total: 0 (0.0)	Acceptable
Blank et al. [22], 2018	Retrospective study, single hospital in Australia	Emergency and elective, laparoscopic and open‡	751	–	During index admission	Total: 2 (0.3)	Acceptable
Farrell et al. [31], 2020	Retrospective study, single hospital trust in the UK	Emergency laparoscopic: 603 Emergency open: 42	645	–	Not defined	Total: 1 (0.2)	Acceptable
Ghirardo et al. [24], 2010	Retrospective study, single hospital in the USA	Emergency and elective laparoscopic: 613 Emergency and elective open: 113	726	–	On day of or day after procedure	Total: 1 (0.1) Emergency laparoscopic: 1 (0.2) Emergency open: 0 (0.0)	Acceptable
Magowan et al. [32], 2020	Retrospective study, single hospital in the UK	Emergency laparoscopic: 282 Emergency converted-to-open: 28 Emergency open: 51	361	361 (100)	Within 30 days of index admission	Total: 0 (0.0)	Acceptable

^*^Quality assessed using the Joanna Briggs Institute Critical Appraisal Checklist for Studies Reporting Prevalence Data [14]: see supplementary material Table S1 for details of these assessments. ‡Number of each type of procedure not specified; †data not subdivided between cholecystectomy and appendectomy therefore these figures include all procedures in the study (*n* = 562); – exact number unknown. *G&S*, group and save; *UK*, United Kingdom; *USA*, United States of America

From the five studies, 4 (0.1%, range 0.0–0.2%) patients in total received perioperative blood transfusion for appendectomy. All articles were deemed to be of an acceptable quality according to the Joanna Briggs Institute Critical Appraisal Checklist for Studies Reporting Prevalence Data (see supplementary material Table S1).

### Patient and operative factors associated with perioperative blood transfusion

A summary of the reported patient and operative factors that may have contributed to perioperative blood transfusion, as well as the timing of transfusion in relation to the index procedure is shown in Table 3. Of the 9807 (2.1%) patients who received a perioperative blood transfusion, information on risk factors and co-morbidities were reported in 45 patients [19, 23, 24, 26, 28, 29]. The main preoperative indications for blood transfusion include cardiovascular co-morbidity 16/45 (35.6%), coagulopathy (including use of anticoagulants) 13/45 (28.9%), moderate anaemia (haemoglobin < 100 g/L) 9/45 (20.0%) and primary haematological malignancy 6/45 (13.3%). The main emergency intraoperative indications for blood transfusion include vascular/solid organ injury and significant intraoperative haemorrhage 21/45 (46.7%) and conversion to open 17/45 (37.8%). From the American Society of Anaesthesiologists (ASA) physical status classification system available amongst the studies, 2/16 (12.5%) were ASA I; 9/16 (56.3%) were ASA II; 4/16 (25.0%) were ASA III and 1/16 (6.3%) were ASA IV patients that received perioperative blood transfusions. Across all the studies, only Usal et al. [19] reported patients that required emergency transfusion (2 patients); other authors suggest there was enough time to obtain new G&S samples prior to transfusing cross-matched blood.

**Table 3:** Summary of studies assessing patient and operative risk factors for blood transfusion where described. *ASA*, American Society of Anaesthesiologists physical status classification system; *Hb*, haemoglobin; *INR*, international normalised ratio; *RR*, relative risk

Authors, year	Operation performed	Patients who received and timing of perioperative blood transfusion, *n*	Summary of reported patient and operative risk factors for transfusion, *n* (% of patients who received a transfusion)
Fong et al. [23], 2021	Cholecystectomy	Total: 12 Preoperative optimisation: 2 Intraoperative: 5 Postoperative within 48 h of procedure: 4 Postoperative > 48 h of procedure: 1 No emergency blood issued nor major vascular injury reported	Moderate preoperative anaemia (Hb < 100 g/L): 7 (58.3) Septic coagulopathy (INR > 1.4): 5 (41.6) Use of oral anticoagulant on admission: 1 (8.3) Conversion-to-open: 6 (50.0; RR compared to completed laparoscopic cholecystectomy: 24.2) Subtotal cholecystectomy: 3 (25.0; RR compared to total cholecystectomy: 10.9)
Ghirardo et al. [24], 2010	Appendectomy	Total: 1 Postoperative day one	Rectus sheath haematoma: 1 (100)
Ghirardo et al. [24], 2010	Cholecystectomy	Total: 5 No emergency blood issued	Moderate preoperative anaemia (Hb < 100 g/L)/primary haematological malignancy: 1 (20.0) Coagulopathy (including use of anticoagulants): 2 (40.0) Open or conversion-to-open: 2 (40.0; RR compared to completed laparoscopic cholecystectomy: 15.2)
Li and Low [26], 2020	Cholecystectomy	Total: 2 Postoperative at 4 and 7 h	ASA II: 1 (50.0) ASA III: 1 (50.0)
Quinn et al. [28], 2011	Cholecystectomy	Total: 48 Preoperative optimisation: 2 Intraoperative: 18 Postoperative: 13 Secondary to re-operation for complications of index procedure: 8 Not documented: 7	Vascular injury: 2 (4.2) Solid organ injury: 6 (12.5) Conversion-to-open: 9 (18.8) ASA III: 2 (4.2) Jaundice: 2 (4.2) Preoperative anticoagulation: 4 (8.3) Primary haematological malignancy: 6 (12.5)
Tandon et al. [29], 2017	Cholecystectomy	Total: 12 All postoperative	ASA I: 2 (16.6) ASA II: 8 (66.6) ASA III: 1 (8.3) ASA IV: 1 (8.3) Significant intraoperative haemorrhage: 10 (83.3) Faecal peritonitis following laparoscopic converted-to-open: 1 (8.3) Postoperative bile leak: 1 (8.3)
Usal et al. [19], 1999	Cholecystectomy	Total: 45 Emergency intraoperative transfusion: 2	Relevant risk factors shown below Major vascular injury: 3 (6.6) Cardiovascular co-morbidity: 16 (35.5) Respiratory co-morbidity: 2 (4.4) Chronic kidney disease: 4 (8.8) Diabetes mellitus: 3 (6.6)

The summary of the overall findings in each study is summarised in Table 4. All authors concluded that G&S testing is unnecessary prior to cholecystectomy and appendectomy, particularly given the low perioperative blood transfusion rate found in each study (range 0.0–2.1%).

**Table 4:** Summary of the findings and reported conclusions of the studies included in the systematic review regarding the need for routine group and save testing. *G&S*, group and save; *MSBOS*, maximum surgical blood ordering schedule

Authors, year	Summary of study findings	Authors conclude that routine preoperative G&S testing may not be necessary
Barrett-Lee et al. [20], 2018	Routine G&S not warranted as low rate of blood transfusion. A more targeted approach required for preoperative G&S and the use of O negative blood is recommended in the rare event of acute haemorrhage from major vessel injury	Yes
Beloeil et al. [21], 2017	Standard ABO blood typing is still routinely prescribed before surgery and anaesthesia. This over-prescription represents a high and unnecessary cost and should therefore be addressed	Yes
Blank et al. [22], 2018	Transfusion rates are low and therefore routine G&S testing for appendectomy is not recommended. Generated site-specific MSBOS is more of an efficient method	Yes
Farrell et al. [31], 2020	Cross-match on an as required basis and use of O negative where urgent blood is required. Huge cost saving with very little impact on demand for O negative blood. Routine G&S testing is unnecessary as rate of transfusion in appendectomy is extremely low	Yes
Fong et al. [23], 2021	Low transfusion rate and patients who did not have a valid G&S sample did not require a transfusion. Patients requiring transfusions were predictable from their pre-operative clinical status—anaemia, sepsis and coagulopathy. Proposed that a highly selective opt-in G&S policy is safe. This would not compromise patient safety and would lead to significant cost savings	Yes
Ghirardo et al. [24], 2010	Routine G&S is not required in absence of preoperative indications. Cholecystectomy is safe with a low transfusion rate. O negative blood has already been screened for the presence of most significant non-ABO antibodies	Yes
Hack-Adams et al. [25], 2015	Patients over investigated and routine G&S testing should be eliminated	Yes
Hamza et al. [11], 2015	Routine G&S is unnecessary	Yes
Li and Low [26], 2020	A preoperative G&S test did not impact management for any patients undergoing laparoscopic cholecystectomy. It should not form part of the routine work-up, although it may still be required for high-risk cases	Yes
Lin et al. [27], 2006	G&S may be safely disregarded	Yes
Magowan et al. [32], 2020	G&S tests are unnecessary and ceasing their requirement as standard may result in significant financial savings. Clinical judgement and the need for various preoperative investigations should be judged on a case-by-case basis by the patient’s surgical and anaesthetic team	Yes
Quinn et al. [28], 2011	Routine use of G&S is not justified. A targeted approach for high risk individuals will reduce demand on blood transfusion service without detriment to patient care	Yes
Tandon et al. [29], 2017	Routine G&S testing is unnecessary. It neither alters the management of severe hypovolaemia secondary to perioperative bleeding, nor does it lead to better outcomes	Yes
Thomson et al. [30], 2016	Abandon preoperative G&S	Yes
Usal et al. [19], 1999	Eliminate routine G&S	Yes

### Financial costs of group and save testing

The financial costs of performing preoperative G&S testing in cholecystectomy and/or appendectomy are summarised in Table 5. The mean reported cost per G&S sample was £18.99 ± 2.87 (median £18.06, range £15.00–£21.30) and the mean cost per year of G&S testing in cholecystectomy and/or appendectomy is £12,908.00 ± 5937.91 (median £12,365.00, range £3,925.00–£22,075.00) in an average-sized hospital in a developed country.

**Table 5:** Summary of the reported and calculated costs of group and save sample per study cohort and per year

Authors, year	Cost per G&S sample (£)	Total cost per study cohort (£)	Total cost per year (£)
Barrett-Lee et al. [20], 2018	17.29	23,131	7710
Farrell et al. [31], 2020	17.50	22,470	7490
Fong et al. [23], 2021	20.00	39,600	15,840
Ghirardo et al. [24], 2010*	21.30	39,050	19,525
Hamza et al. [11], 2015	17.24	13,280	13,280
Li and Low [26], 2020	15.00	22,075	22,075
Magowan et al. [32], 2020	25.40	18,346	18,346
Quinn et al. [28], 2011	20.00	80,140	11,449
Thomson et al. [30], 2016	18.37	7850	3925
Usal et al. [19], 1999*	17.75	56,658	9443

^*^Converted from $ to pounds. *G&S*, group and save

## Discussion

We evaluated the existing published literature on the rate of perioperative blood transfusion and the need for G&S testing prior to cholecystectomy and appendectomy. Our review demonstrates that preoperative G&S testing is being performed nationally and internationally, whether as a mandatory policy in many hospitals [23, 24, 29, 32, 34], or owing to limited available guidance.

We found an extremely low risk of blood transfusion rate of 2.1% across the 15 studies, with only two patients [19] requiring intraoperative emergency transfusions. It was noted that there was enough time to obtain new G&S samples prior to transfusing cross-matched blood in all other transfused cases. It has previously been reported that age, gender, ASA and body mass index do not appear to influence bleeding risk [35]. However, in this review, we were unable to confidently quantify the risk of transfusion associated with these factors due to the limited number of patients that underwent a perioperative transfusion.

In the available literature, there is no strong evidence supporting the routine use of preoperative G&S testing prior to cholecystectomy and appendectomy. All 15 studies in this systematic review concluded that routine preoperative G&S testing was not necessary. The benefits of not performing compulsory G&S testing include fewer emergency and elective theatre delays, and reduced demand on staff, blood transfusion and phlebotomy departments with financial implications. The studies did not demonstrate positive outcomes for patients in favour of preoperative G&S testing, nor any negative outcomes in patients who did not have a G&S test. However, it must be noted that the requirement for G&S testing should depend on the rate of blood transfusion for a particular surgery in an individual centre. Ideally, the relevant department should audit the rates of blood transfusion per surgery every year and decide on whether G&S testing is absolutely necessary for that specific procedure.

This systematic review confirms that there is limited national and international guidance specifically relating to routine preoperative G&S testing in cholecystectomy and appendectomy. In 2012, SFAR [2, 21] issued guidelines in order to rationalise and reduce preoperative tests. SFAR do not recommend blood typing when there is a low risk of transfusion [34], for example less than 5%. These guidelines were endorsed by 17 surgical and medical scientific societies that have promoted their use. They recommended that the following items were included in a preoperative questionnaire: tendency for prolonged/unusual bleeding, tendency to develop ecchymoses/bruising/haematomas, prolonged bleeding after tooth extraction, major bleeding after surgery, family history, and in women, menorrhagia or postpartum haemorrhage [21]. In 2016, NICE also published a preoperative guidance in order to standardise the process of preoperative investigation across the UK [1]. However, G&S testing was excluded from these guidelines. This was deemed to be a clinical decision dependent on operative severity and the likelihood of blood loss. Similarly, the British Association of Day Surgery (BADS) and the Association of Anaesthetists of Great Britain and Ireland (AAGBI) have a document on day-case surgery which does not specifically mention the role of preoperative G&S [36].

Fong et al. [23] suggested that the patients that required a blood transfusion were predictable from their preoperative clinical status and risk factors, and therefore a highly selective opt-in policy is safe and would not compromise patient safety. Routinely sending two G&S samples prior to cholecystectomy and appendectomy may be an unnecessary use of resources. Ghirado et al. [24] similarly suggested that the risk of transfusion appears to be related to pre-existing medical conditions, such as anticoagulation treatment and preoperative anaemia, and a targeted approach would be more beneficial. Beloeil et al. [21] reported the largest cohort of cholecystectomy patients included in this review, of which 37.2% of patients underwent preoperative G&S testing. This study also assessed the need for testing in thyroidectomy, lumbar discectomy and breast surgery. They concluded that routine G&S testing needs to be addressed as it leads to a high and unnecessary cost with no clinical impact. Li and Low [26] has successfully removed G&S testing, in the absence of haemoglobinopathies and risk factors for red cell antibodies, from preoperative screening with no resultant adverse consequences.

There is a perception from anaesthetic and surgical staff that there is an increased risk of major haemorrhage during laparoscopic surgery [31]. We have confirmed findings from other studies that transfusion rates are low and major vascular injury rarely occurs in laparoscopic surgery. In a large meta-analysis, Larobina and Nottle [37] found the incidence of major vascular injury to be 0.044% in 760,890 closed-entry laparoscopies and 0% in 22,465 open-entry laparoscopies. Another meta-analysis estimated the bleeding complication rate to be between 0.54 and 1.05% [38]. If such complications were to arise, the situation would likely necessitate activation of a haemorrhage protocol and immediate procurement of unmatched blood products, such as O negative blood, platelets, and fresh frozen plasma. Waiting the twenty minutes required to obtain cross-matched blood, even if a preoperative G&S sample has been taken, is likely to be to the patient’s detriment [24].

Errors from G&S testing resulting in sample rejection from the blood bank have also been described in the literature [20]. These include incomplete details, sample haemolysis, duplicate sample, incorrect details, unsuitable specimen and details not handwritten. Preoperative G&S samples that are invalid lead to delays in emergency and elective operating lists whilst new samples need to be taken. In addition to the inefficient running of these lists, patients may be exposed to further invasive procedures and conflict can arise between surgical and anaesthetic teams regarding the perceived necessity of these tests.

Based on the evidence, G&S testing per sample costs approximately £15.00 to £21.30, ranging from £3,925.00 to £22,075.00 per year for an average-sized hospital in a developed country. Although not a particularly expensive test, with over 34,000 appendectomies and 65,000 cholecystectomies performed in the National Health Service in 2019–2020, for example [39], the associated burden on junior doctors’ and phlebotomists’ workload needs to be considered. Taking a more selective approach when choosing in whom to perform G&S testing could result in significant savings and better use of resources for the health service.

### Limitations

There are several limitations that must be taken into account when interpreting the findings of this systematic review. Although G&S testing is routinely carried out in many centres nationally and internationally, we were only able to discover a relatively small number of studies relating to the requirement of routine use of preoperative G&S testing. The majority of the data from this review is extracted from two nationwide French datasets [21] and in general developed countries. In addition, the specific number of patients that underwent G&S testing was not extractable in a few of the studies and the precise definition of perioperative blood transfusion was variable ranging from on the day of the procedure to within 30 days of the index admission. Nevertheless, we have managed to evaluate the need for routine G&S testing to help provide guidance for surgical, anaesthetic and nursing staff, and identify the risk factors of perioperative transfusion associated with cholecystectomy and appendectomy. As perioperative blood transfusion rates are rare, larger retrospective studies of G&S testing in surgery along with the evaluation of specific indicators for perioperative transfusion are required to be adequately powered.

## Conclusion

This is the first systematic review assessing the need for preoperative G&S screening, which provides evidence-based guidance for surgical, anaesthetic and nursing teams. Based on the available literature, routine G&S testing is not necessarily required for all patients undergoing cholecystectomy or appendectomy. There is no strong evidence to suggest that routine G&S screening benefits patient outcomes and safety. G&S testing should be requested on a patient case-specific basis with discussions between the anaesthetist and surgeon. High-risk criteria that we could identify for blood transfusion include septic coagulopathy, anticoagulation treatment, preoperative anaemia, cardiovascular co-morbidity, antibodies on a previous sample and a history of haematological malignancy. We therefore recommend that G&S testing is reserved for patients with these risk factors prior to cholecystectomy or appendectomy. Larger retrospective studies of the necessity of G&S testing in surgery are required for further evaluation of preoperative risk factors for perioperative blood transfusion.

## Supplementary Information

Below is the link to the electronic supplementary material.

Supplementary file1 (DOCX 17 KB)
