# Supplementary material for: Requirement of preoperative blood typing for cholecystectomy and appendectomy: a systematic review
Source: Langenbecks Arch Surg. 2022 Jul 2;407(6):2205–16. doi: 10.1007/s00423-022-02600-x (PMC9468044; doi:10.1007/s00423-022-02600-x)
Supplement: Supplementary file 1 — Supplementary file1 (DOCX 17 KB) [file 423_2022_2600_MOESM1_ESM.docx]

**Supplementary material**

| **Author, year** | **Study timeline** | **Study type** | **Single centre** | **Study location** |
| --- | --- | --- | --- | --- |
| Barrett-Lee *et al*. [20], 2018 | January 2014 - December 2016 | Retrospective | Yes | UK |
| Beloeil *et al*. [21], 2017 | 2012 - 2015 | Retrospective | No  *(multicentre)* | France |
| Blank *et al*. [22], 2018 | May 2012 - April 2017 | Retrospective | Yes | Australia |
| Farrell *et al*. [31], 2020 | 3 year period | Retrospective | Yes | UK |
| Fong *et al*. [23], 2021 | March 2014 - October 2016 | Retrospective | Yes | UK |
| Ghirardo *et al*. [24], 2010 | July 2005 - July 2007 | Retrospective | Yes | USA |
| Hack-Adams *et al*. [25], 2015 | December 2013 – January 2014 | Retrospective | Yes | UK |
| Hamza *et al.* 113], 2015 | 2002 - 2011 | Retrospective | No  *(3 hospitals within Trust)* | UK |
| Li and Low [26], 2020 | January 2016 - January 2017 | Retrospective | Yes | UK |
| Lin *et al*. [27], 2006 | December 2004 - March 2005 | Retrospective | Yes | Taiwan |
| Magowan *et al*. [32], 2020 | April 2018 - April 2019 | Retrospective | Yes | South Wales, UK |
| Quinn *et al*. [28], 2011 | January 1998 - February 2005 | Retrospective | Yes | UK |
| Tandon *et al*. [29], 2017 | 2010 - 2014 | Retrospective | Yes | UK |
| Thomson *et al*. [30], 2016 | April 2012 - March 2014 | Retrospective | Yes | UK |
| Usal *et al*. [19], 1999 | January 1990 - December 1996 | Retrospective | Yes | USA |

**Table S1.** List of studies included in the systematic review. UK, United Kingdom; USA, United States of America.

| **Authors, year** | **Q1. Was the sample frame appropriate to address the target population?** | **Q2. Were study participants sampled in an appropriate way?** | **Q3. Was the sample size adequate?** | **Q4. Were the study subjects and the setting described in detail?** | **Q5. Was the data analysis conducted with sufficient coverage of the identified sample?** | **Q6. Were valid methods used for the identification of the condition?** | **Q7. Was the condition measured in a standard, reliable way for all participants?** | **Q8. Was there appropriate statistical analysis?** | **Q9. Was the response rate adequate, and if not, was the low response rate managed appropriately?** | **Total number of criteria met** | **Quality** |
| --- | --- | --- | --- | --- | --- | --- | --- | --- | --- | --- | --- |
| Barrett-Lee *et al*. [20], 2018 | Yes | Yes | Yes | Yes | Yes | Yes | Yes | Yes | Yes | 9 | Acceptable |
| Beloeil *et al*. [21], 2017 | Yes | Yes | Yes | Yes | Yes | Yes | Yes | Yes | Yes | 9 | Acceptable |
| Blank *et al*. [22], 2018 | Yes | Yes | Yes | Yes | Yes | Yes | Yes | Yes | Yes | 9 | Acceptable |
| Farrell *et al*. [31], 2020 | Yes | Yes | Yes | Yes | Yes | Yes | Yes | Yes | Yes | 9 | Acceptable |
| Fong *et al*. [23], 2021 | Yes | Yes | Yes | Yes | Yes | Yes | Yes | Yes | Yes | 9 | Acceptable |
| Ghirardo *et al*. [24], 2010 | Yes | Yes | Yes | Yes | Yes | Yes | Yes | Yes | Yes | 9 | Acceptable |
| Hack-Adams *et al*. [25], 2015 | Yes | Yes | No | Yes | Yes | Yes | Yes | Yes | Yes | 8 | Acceptable |
| Hamza *et al.* [11], 2015 | Yes | Yes | Yes | Yes | Yes | Yes | Yes | Yes | Yes | 9 | Acceptable |
| Li and Low [26], 2020 | Yes | Yes | Yes | Yes | Yes | Yes | Yes | Yes | Yes | 9 | Acceptable |
| Lin *et al*. [27], 2006 | Yes | Yes | No | Yes | Yes | Yes | Yes | Yes | Yes | 8 | Acceptable |
| Magowan *et al*. [32], 2020 | Yes | Yes | No | Yes | Yes | Yes | Yes | Yes | Yes | 8 | Acceptable |
| Quinn *et al*. [28], 2011 | Yes | Yes | Yes | Yes | Yes | Yes | Yes | Yes | Yes | 9 | Acceptable |
| Tandon *et al*. [29], 2017 | Yes | Yes | Yes | Yes | Yes | Yes | Yes | Yes | Yes | 9 | Acceptable |
| Thomson *et al*. [20], 2016 | Yes | Yes | No | Yes | Yes | Yes | Yes | Yes | Yes | 8 | Acceptable |
| Usal *et al*. [19], 1999^*^ | Yes | Yes | Yes | Yes | Yes | Yes | Yes | Yes | Yes | 9 | Acceptable |

**Table S2.** Joanna Briggs Institute Critical Appraisal Checklist for Studies Reporting Prevalence Data.
